# Evaluating differences in respiratory motion estimates during radiotherapy: a single planning 4DMRI versus daily 4DMRI

**DOI:** 10.1186/s13014-021-01915-1

**Published:** 2021-09-26

**Authors:** Duncan den Boer, Johannes K. Veldman, Geertjan van Tienhoven, Arjan Bel, Zdenko van Kesteren

**Affiliations:** Department of Radiotherapy, Amsterdam University Medical Centers, Meibergdreef 9, 1105 AZ Amsterdam, The Netherlands

**Keywords:** Radiotherapy, 4DMRI, Respiratory motion, Abdominal cancer, Outlier rejection

## Abstract

**Background:**

In radiotherapy, respiratory-induced tumor motion is typically measured using a single four-dimensional computed tomography acquisition (4DCT). Irregular breathing leads to inaccurate motion estimates, potentially resulting in undertreatment of the tumor and unnecessary dose to healthy tissue. The aim of the research was to determine if a daily pre-treatment 4DMRI-strategy led to a significantly improved motion estimate compared to single planning 4DMRI (with or without outlier rejection).

**Methods:**

4DMRI data sets from 10 healthy volunteers were acquired. The first acquisition simulated a planning MRI, the respiratory motion estimate (constructed from the respiratory signal, i.e. the 1D navigator) was compared to the respiratory signal in the subsequent scans (simulating 5–29 treatment fractions). The same procedure was performed using the first acquisition of each day as an estimate for the subsequent acquisitions that day (2 per day, 4–20 per volunteer), simulating a daily MRI strategy. This was done for three outlier strategies: no outlier rejection (NoOR); excluding 5% of the respiratory signal whilst minimizing the range (Min95) and excluding the datapoints outside the mean end-inhalation and end-exhalation positions (MeanIE).

**Results:**

The planning MRI median motion estimates were 27 mm for NoOR, 18 mm for Min95, and 13 mm for MeanIE. The daily MRI median motion estimates were 29 mm for NoOR, 19 mm for Min95 and 15 mm for MeanIE. The percentage of time outside the motion estimate were for the planning MRI: 2%, 10% and 32% for NoOR, Min95 and MeanIE respectively. These values were reduced with the daily MRI strategy: 0%, 6% and 17%. Applying Min95 accounted for a 30% decrease in motion estimate compared to NoOR.

**Conclusion:**

A daily MRI improved the estimation of respiratory motion as compared to a single 4D (planning) MRI significantly. Combining the Min95 technique with a daily 4DMRI resulted in a decrease of inclusion time of 6% with a 30% decrease of motion. Outlier rejection alone on a planning MRI often led to underestimation of the movement and could potentially lead to an underdosage.

*Trial registration*: protocol W15_373#16.007

**Supplementary Information:**

The online version contains supplementary material available at 10.1186/s13014-021-01915-1.

## Introduction

Radiotherapy for upper abdominal cancer, such as pancreatic, esophageal and gastric cancer, is challenging due to poor contrast between tumor and other soft tissue on planning CT scans, as well as respiratory induced motion of both tumor and organs. The poor soft-tissue contrast in the abdominal region on planning CT can be overcome by adding MRI images. This decreases delineation variation [1–6] and is becoming common practice in the clinic [7]. The respiratory motion is often dealt with by obtaining a 4DCT and delineating the tumor in the different respiration phases, and subsequently combining these delineations to generate an Internal Target Volume (ITV) [8, 9]. This ITV then acts as the target volume for the entire treatment. Similar to 4DCT, 4DMRI acquisitions can be employed for the delineation of the tumor, capturing the tumor motion [10–15]. Irregular breathing patterns during acquisition (e.g. caused by coughs or hiccups) can lead to image artefacts, both decreasing the image quality as well as the accuracy of the reconstructed respiratory motion. This motion may be overestimated which could potentially lead to the unnecessary exposure of healthy tissue or underestimated leading to insufficient tumor coverage.

Recent work has shown that the image quality of such acquisitions improved by applying an outlier rejection strategy [16]. Potentially, an outlier rejection strategy can give an estimate of the reconstructed respiratory motion that is more representative for the treatment, than one without rejection of outliers. Outlier rejection leads to a smaller motion estimate which decreases the volume of irradiated healthy tissue, however increasing the risk of undertreatment. In current clinical practice (typically employing 4DCT) no outlier rejection is applied. Some dedicated systems such as Cyberknife take the respiratory motion into account during treatment delivery [17].

A recent development in the field is the clinical availability of MR-Linac (MRL) systems capable of MR-guided radiotherapy (MRgRT) [18–20] that have been used for daily adaptive treatments for several abdominal tumor sites [21–27]. The MRL is designed to deal with interfraction changes through daily adaptation of the treatment plan with incorporation of the daily anatomy. One can also image during the delivery of the treatment and apply a gating strategy, in which the beam is switched off if the tumor moves outside of the Planning Target Volume (PTV) boundaries, hence dealing with intrafraction motion [18, 28]. With this or other online image guided techniques respiratory motion does not have to be included as an expansion of the PTV or for the generation of an ITV. In the case of a small difference in contrast between tumor and tissue for the MRI sequence used for gating, it can be challenging to discern the tumor on the intra-treatment acquisitions, and currently not all clinically available systems have this gating feature. In those cases it is still necessary to deal with the respiratory motion by generating an appropriate target volume, for example by obtaining a daily 4DMRI and/or applying a strategy to decrease the breathing motion, for example by breath holding [28].

For the treatment of liver cancer, it has been shown that it is feasible to obtain a 4DMRI at the start of each fraction [29] to aid with patient positioning, and with treatment of kidney cancer it shows potential also for intrafraction adaptation [30]. Besides the differences in tumor position, there is often a day to day difference in the magnitude of the respiratory-induced motion. This needs to be dealt with during treatment, for example by adapting the margins. Obtaining a daily pre-treatment 4DMRI gives one the option to estimate the motion-of-the-day and adapt the target volume accordingly. These insights could prove valuable especially in the context of MR-guided radiotherapy as acquiring daily MRI images is part of the standard workflow. To the best of our knowledge, this is so far not implemented clinically nor studied in detail.

In this study we evaluated if a daily pre-treatment 4DMRI led to a significant improvement for the estimation of respiratory-induced motion of a subsequent acquisition (as a simulated treatment fraction) as compared to a single planning 4DMRI. Additionally we evaluated the effect of outlier rejection on this motion estimate, for both these strategies. This evaluation was done by analyzing respiratory-induced motion of the right hemidiaphragm of the navigator in series of 4DMRI data of healthy volunteers, as a simulation data set for a radiotherapy treatment.

## Materials and methods

### Volunteers

**Fig. 1:**
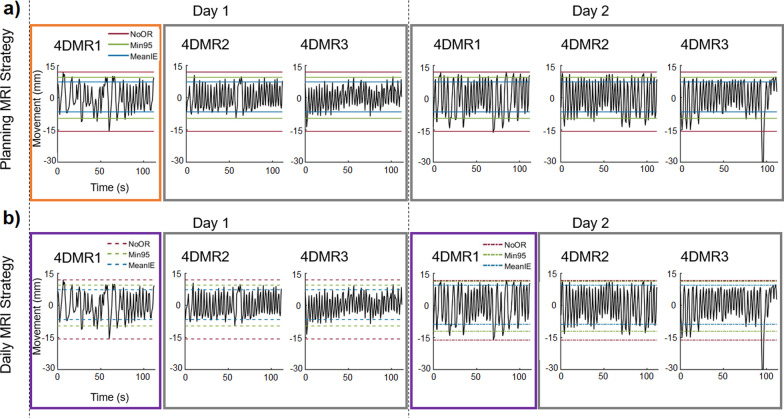
Overview of scanning sessions. For each volunteer, three consecutive 4DMRI scans were obtained during a session, 4DMR1, 4DMR2 and 4DMR3 (sessions always on separate days). All volunteers had at least two sessions, while Volunteer 1 and Volunteer 2 had ten sessions. Respiratory induced motion estimates were generated from the 1D navigator signal of the right hemidiaphragm of a reference MRI for the outlier rejection strategies NoOR, MeanIE and Min95 (see text for details). **a** For the planning MRI strategy the first obtained scan was used as a reference (mimicking a planning MRI), indicated by the orange box. The motion estimates were evaluated for all subsequent scans (indicated by the grey boxes, simulating treatment fractions). **b** For the daily MRI strategy the first scan of each session (indicated by the purple box) was used as a reference and the motion estimates were evaluated on the two following scans of the same day (indicated by the grey boxes, simulating treatment fractions)

From December 2018 till June 2019, ten healthy volunteers (six male, four female, median 35 years, range 20–55 years) participated in the study. They all gave written informed consent in accordance with the medical ethical regulations at our institute. Eight volunteers came for two and two volunteers (Volunteer 1 and Volunteer 2) for ten sessions, with at least a week between. These longer series gave an indication for the intrasubject variation as compared to the intersubject variation. During a session three consecutive 4D acquisitions were obtained (4DMR1, 4DMR2, 4DMR3, see Fig. 1). Eight volunteers with two sessions of three scans each led to 48 scans; two volunteers with ten sessions of three scans each (with 1 acquisition failure) led to 59 scans coming to a total of 107 scans. For the planning MRI strategy these acquisitions simulate a planning MRI (the first acquisition, Fig. 1a) and treatment fractions (the subsequent acquisitions, Fig. 1a). For the daily MRI strategy these acquisitions simulate a pre-treatment daily acquisition (the first acquisition of a session, Fig. 1b) and treatment fractions (the subsequent acquisitions Fig. 1b).

### Image acquisition

Images were acquired on a 3.0 T MRI scanner (Ingenia 3.0 T, Philips Healthcare, Best, The Netherlands) using a T2-weighted single-shot turbo spin echo sequence with a field of view of 400 × 200 (superior-inferior × right-left) mm^2^, repetition time of 6061 ms, echo time of 50 ms, and a flip angle of 90 degrees. Receiver bandwidth was 555.9 Hz/pixel and sensitivity encoding (SENSE) factor was 4. The sequence and its parameters had been optimized for upper abdominal imaging. Each volume consisted of 11 coronal 2D slices and was acquired repetitively 60 times, i.e., 60 dynamics, during free breathing. The acquired 2D slices had a resolution of 0.78 × 0.78 mm^2^ in-plane and 5 mm slice thickness and were acquired in an interleaved fashion. Image acquisition was interleaved with a 1D navigator, located on the top of the right hemidiaphragm, yielding the diaphragm position every 551 ms (corresponding to the slice acquisition time). This navigator was used as a respiratory signal surrogate, associating each acquired 2D image with a respiratory state, used for 4DMRI binning, outlier rejection and strategy evaluation (see below). To correct for geometrical distortions, the 2D gradient non-linearity corrections as provided by the vendor were used. The total scan time was 6 min, obtaining 660 images per data set.

**Fig. 2:**
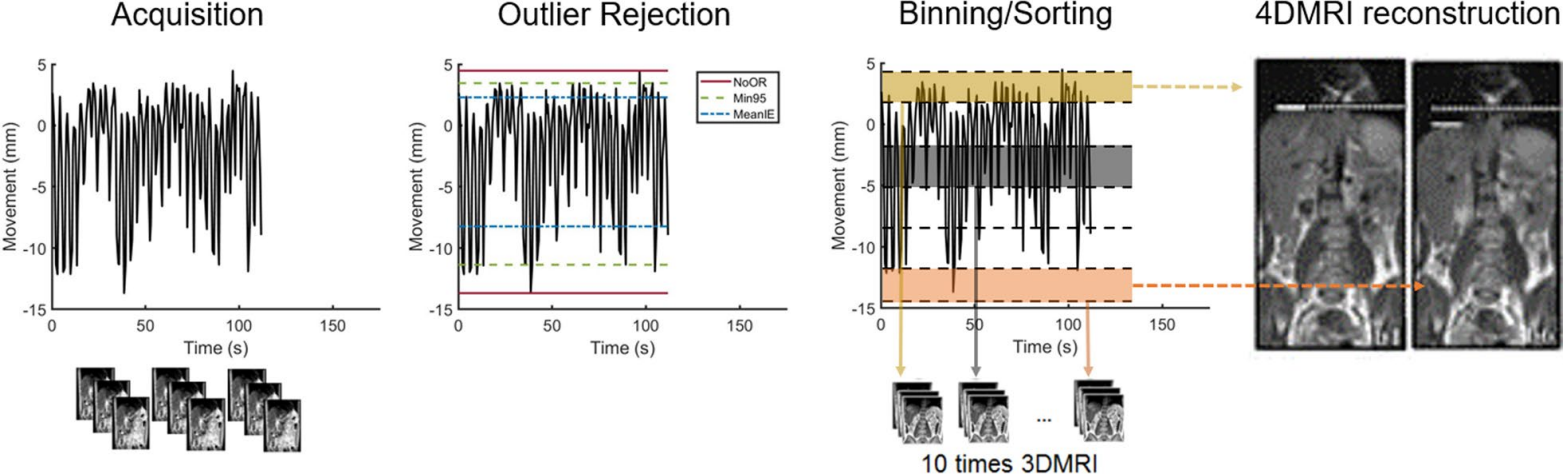
4DMRI reconstruction. During acquisition, motion of the diaphragm is recorded using a 1D navigator, which is acquired simultaneously with the images. Afterwards, outlier rejection is applied using three different strategies: NoOR (Red), Min95 (Green) and MeanIE (Blue). The resulting respiratory signal is binned into ten amplitude bins and the corresponding images are sorted based on their bin and slice number. From each amplitude bin, a 3D volume can be reconstructed. The resulting 4DMRI consists of ten respiratory-correlated MRI volumes with each eleven slices

Data processing (navigator extraction, binning, sorting, outlier rejection, see Fig. 2) was performed using a home-built algorithm in MatLab (MatLab R2018b, The Math Works Inc, Natick, MA).

### Binning and outlier rejection

The respiratory signal was used to bin the breathing cycle in amplitude bins, according to the position of the respiratory surrogate. Ten bins were defined, where the bins at end-inhalation and end-exhalation level were half the size of the other bins [16]. Irregular breathing can have a large effect on the quality of binning and the quality of reconstructed images. Amplitude binning is more robust for this effect, especially in combination with outlier rejection [16].

For this study we applied three outlier rejection strategies before amplitude binning. (1) No outlier rejection (denoted as NoOR), (2) Discarding outliers outside upper and lower inclusion thresholds that were chosen such that 95% of the acquired images were included (i.e., 627 images), whilst minimizing the distance between thresholds (denoted as Min95 [16]), and (3) Discarding outliers outside the mean end-inhalation and mean end-exhalation levels (denoted as MeanIE [31]).

After outlier rejection, ten bins were defined, where the bins at end-inhalation and end-exhalation level were half the size of the other bins. The 2D images were sorted according to the navigator position. In cases that multiple images of one slice position were assigned to the same bin, the image with the median diaphragm position was selected [16]. See Additional file 1: Fig A1 for an example 4DMRI.

### Evaluation motion estimate

For the planning MRI strategy (Fig. 1a) the motion estimates were generated for all three outlier rejection strategies with the first MRI acquisition as a reference for all subsequent scans and for the daily MRI strategy with the first scan of a daily session (Fig. 1b) as reference for the scans of the same day. These motion estimates were constructed from the maximum peak to peak distance of the respiratory trace of the reference 4DMRI after outlier rejection. In practice, these motion estimates will not perfectly fit the breathing amplitude during the subsequent simulated treatment fractions due to irregularity of the breathing of the patient. To mimic position verification during treatment, the average position of the signal during the simulated treatment fraction was shifted to match the average position of the respiratory signal of the reference 4DMRI.

Two suboptimal scenarios were possible: the motion estimate was too small compared to the breathing amplitude in the simulated treatment fraction (which would lead to target undertreatment), or the motion estimate was too large (which would lead to irradiating healthy tissue). To evaluate these scenarios we analyzed motion estimates for the simulated treatment fractions in the following way:

1. To quantify the underestimation of the motion estimate, two approaches were used. A) The relative time that the respiratory signal of the simulated treatment fraction was outside the motion estimate was determined. This is the relative time that the target would be partly missed during irradiation if no additional margin had been applied. Note that for the simulated treatment fraction, no outlier rejection was applied, only for the construction of the motion estimate during the reference MRI acquisition. B) The distance of the navigator position outside the motion estimate was calculated per position for each of the simulated treatment fractions. For each volunteer the relative frequencies of these distances were determined (with a resolution of 0.5 mm) and histograms were constructed from the data of all volunteers, showing the median value and the range. The relative frequency at a certain distance indicates how much time the navigator was that far outside the motion estimate. For this analysis all navigator points outside the motion estimate were included, as opposed to only mean or maximum inhale and exhale values for each fraction (see below). From these histograms, we determined the distance to the motion estimate within which 95% and 99% of the data was included.
2. To quantify the overestimation of the motion estimate, two approaches were used (see also Additional file 1: Fig. A2). (A) The maximum observed motion during a simulated treatment fraction was analyzed and the distance to the thresholds of the motion estimate was determined. (B) The distances of the mean end-inhalation and end-exhalation level to the higher and lower threshold of the motion estimate were determined [32, 33]. Both these strategies gave two values per simulated treatment scan (a distance for the inhale and exhale threshold).

A paired two-sided Wilcoxon’s signed-rank test was used to test for significance in differences between the NoOR, Min95 and MeanIE outlier rejection strategies for the scans within a strategy (either planning MRI or daily MRI). For the comparison of data of NoOR, MeanIE and Min95 for the planning MRI strategy (N = 97 simulated treatment fractions; Total 107 scans of which 10 reference scans) to the daily MRI strategy (N = 71 simulated treatment fractions; Total 107 scans, of which 36 reference scans) a two-sided Wilcoxon rank sum test was used. For both types of tests a significance level α = 0.05 was applied.

## Results

**Fig. 3:**
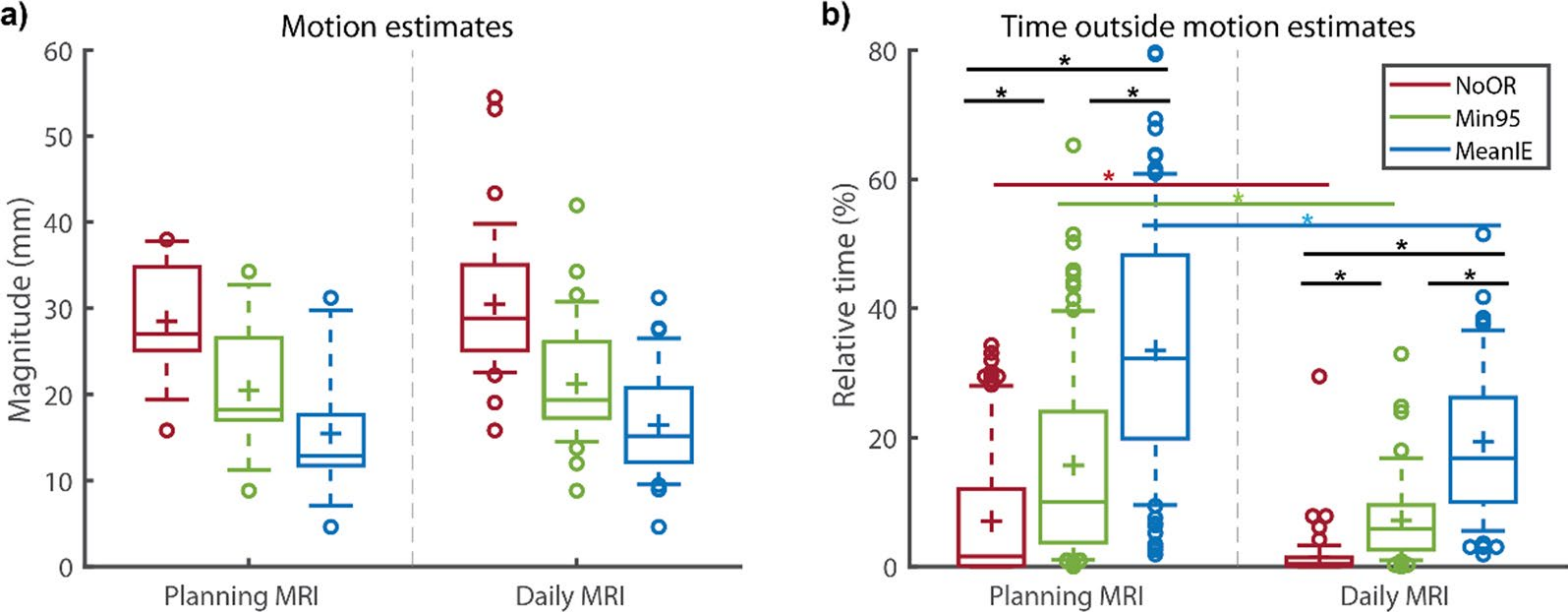
Motion estimates and time outside the estimates. **a** Motion estimates for NoOR, Min95 and MeanIE for all reference scans of all volunteers for the planning MRI strategy (N = 10) and the daily MRI (N = 36). Boxes: median value (line), mean (cross) and lower and higher quartiles; whiskers: lowest and highest data point in the 9–91% interval, the outliers are indicated by circles. * indicates statistically difference with α = 0.05. **b** The percentage of time the simulated fractions were outside the estimated motions (as depicted in a). For planning MRI: N = 97 included scans; for daily MRI strategy N = 71 included scans

The motion estimates were determined for the planning MRI (N = 10, first acquisition of each volunteer) and for the daily MRI (N = 36, first acquisition of each session for each volunteer) strategies, and the results are depicted in Fig. 3a. The values were statistically significantly different with NoOR > Min95 > MeanIE for both strategies. For the planning MRI the motion estimates for NoOR were 27 mm, 16–38 mm (median, range), for Min95 18 mm, 9–34 mm and for MeanIE 13 mm, 5–31 mm. For the daily MRI the motion estimates (median, range) were 29 mm, 16–55 mm for NoOR and 19 mm, 9–42 mm for Min95 and 15 mm, 5–31 mm for MeanIE. The motion estimates for the planning MRI strategy and the corresponding values for the daily MRI strategy did not differ significantly. For individual values of the motion estimates per volunteer see Additional file 1: Fig. A3. The intrasubject variation of the maximum observed respiratory motion, as observed in the longer series of Volunteer 1 and 2, differed, but were in the same range as the intersubject variations (Additional file 1: Fig. A4).

The percentage of time outside the motion estimate had an opposite trend to the motion estimates; for both strategies NoOR < Min95 < MeanIE, as can be seen in Fig. 3b.

For the planning MRI strategy, the percentage of time outside the motion estimate of the NoOR was 2%, 0–34% (median, range), with a maximum median value of 29% for Volunteer 7 (based on 5 simulated treatment fractions). The median value for Min95 was 10%, 0–65%, with the worst case for Volunteer 3 where the respiratory signal was outside the motion estimate for a simulated fraction for 65% of the time. The percentage of time outside the motion estimates was significantly higher for MeanIE, 32%, 2–80%, with also an extreme value for Volunteer 3, with a single simulated treatment fraction with 80% outside the motion (see Additional file 1: Fig. A5).

For the daily MRI strategy these values were all significantly lower than the corresponding values for the planning MRI, 0%, 0–30% for NoOR, 6%, 0–33% for Min95, 17%, 2–70% for MeanIE; median and range. Whereas this trend was observed for the total of scans, this was not always the case for individual volunteers. For example the MeanIE of Volunteer 9 had more time outside the motion estimates for the daily MRI than for the planning MRI, due to a large respiratory motion during the planning MRI, as compared to subsequent simulated treatment fractions.

Histograms of the observed motion in the simulated treatment fractions outside the motion estimates are depicted in Fig. 5 (relative frequencies vs specific distances). The distance to the motion estimate within which 99% of the data was included was 3 mm and 0 mm for NoOR for the planning MRI and daily MRI respectively. For Min95 these distances were 5 mm and 4 mm and for MeanIE 8 mm and 6 mm. For NoOR there was always more than 95% of the data already within the motion estimate. For Min95 these distances were 3 mm and 0.5 mm, and for MeanIE 6 mm and 4 mm for planning MRI and daily MRI respectively.

The difference between the motion estimates of NoOR, Min95 and MeanIE for planning MRI and daily MRI and the mean end-inhalation and end-exhalation levels of the simulated treatment fractions is depicted in Additional file 1: Fig. A6a. The motion estimates were higher than the observed values for NoOR and Min95, with values (median, range) for the planning MRI, NoOR: 5 mm, − 5–19 mm; MeanIE: 0 mm, − 9–15 mm; Min95: 2 mm, − 7–16 mm and for the daily MRI, NoOR: 6 mm, − 4–37 mm; MeanIE: 0 mm, − 6–15 mm. Min95: 3 mm, − 5–26 mm, with a positive value meaning a higher motion estimate and a negative value a lower motion estimate. The results of a similar analysis, looking at the differences between the motion estimates and the maximum end-inhalation and end-exhalation levels of a simulated treatment fraction can be found in Additional file 1: Fig. A6b. In this case the median and range for the planning MRI were NoOR: 0 mm − 41–15 mm, MeanIE: − 7 mm, − 52–8 mm, Min95: − 4 mm − 50–10 mm; and for the daily MRI, NoOR: 0 mm, − 38–32 mm; MeanIE: − 5 mm − 47–7 mm; Min95: − 3 mm, − 44–9 mm.

## Discussion

4DMRI volunteer data was used to simulate a multi-fraction radiotherapy treatment to evaluate the efficacy of constructed motion estimates, with and without outlier rejection. These motion estimates were evaluated for their efficacy to estimate the respiratory motion in subsequent fractions in the case of a single planning MRI and in the case of a daily MRI, by determining the relative time the motion was outside the motion estimate and analyzing the distances between the observed motion and motion estimates. For a daily MRI the respiratory motion of the simulated treatment fractions was within the motion estimates for a statistically significant larger percentage of the time than for the planning MRI strategy.

Estimating the respiratory motion based on a single 4D acquisition leads to a large percentage of time that the motion during the simulated treatment fractions is outside the estimated motion, with or without outlier rejection; sessions were observed where this was the case 80% (MeanIE) and over 30% of the time (NoOR). A daily 4DMRI acquisition leads to a better estimation during the treatment fraction with the respiratory motion being less time outside the motion estimate and the deviations between this respiratory motion and the motion estimate being smaller.

The Min95 strategy was designed to include the signal 95% of the time, accepting 5% time that the signal was outside this motion estimate. The rationale behind this was that breathing irregularities occur only a small percentage of the time but can have a large effect on the peak to peak motion amplitude, and with this strategy effectively mitigated. However, for the planning MRI strategy, the median value of the percentage of time outside the motion estimate of the simulated fractions following the planning MRI was 10%, double the value of 5%. The other, more strict outlier rejection, which had been researched previously [31], MeanIE, had an even higher value of 32% that the motion was underestimated. This shows that outlier rejection alone does not mitigate the effects of the variation in respiratory signal and that a too strict strategy can lead to a large underestimation of the motion. Other outlier strategies exist [4, 34, 35], but based on our results we do not expect these to better mitigate the effect of respiratory signal variation during a radiation therapy treatment series than the strategies in our study as they were typically designed with a different goal, i.e. improve image quality and reduce image artefacts.

No outlier rejection (NoOR, which is now current clinical practice, where a planning 4DCT is used to construct an ITV) in which the complete peak to peak motion of the planning MRI was taken as the motion estimate performed better than Min95 and MeanIE in terms of inclusion time, with a median value of 2% that the respiratory signal was outside the motion estimates. While this median value was acceptable, the maximum observed value of relative time outside the motion estimate was 34% for a simulated treatment fraction, due to breathing irregularities which were not reproducible between time of pre-treatment scanning and during subsequent acquisitions.

When the planning MRI strategy was compared to the daily MRI strategy, it was seen that the daily strategy performed significantly better for all three outlier rejection strategies, with better median values and smaller ranges for the time the simulated fractions were outside the motion estimates. This showed that the intrafraction variation was smaller than the interfraction variation in the respiratory motion. This suggests that it can be beneficial to determine the “motion of the day” and account for this for each treatment fraction [9].

Van de Lindt et al. [29] also suggested a daily pre-beam 4DMRI and showed that this was feasible for MRL patients that were treated for liver tumors. However, they used the 4DMRI to generate an averaged mid-position that was subsequently used for positioning but did not use the information gained by this 4DMRI to estimate the motion for that fraction and incorporate that in the treatment. Our results indicate that this could be beneficial in terms of the time that the motion is within the estimated motion. However, the interval in our study per session is approximately 18 min, which corresponds to a typical conventional linac timeslot but timeslots at an MR-Linac are generally longer by a factor of two or more. More variation in respiratory motion could for example occur due to patient discomfort and anxiety over such a longer time interval.

Interestingly, when the values for the motion estimates were compared of the planning MRI strategy to the daily MRI strategy, the corresponding values were the same for NoOR, Min95 and MeanIE. This suggests that accounting for the motion of the day will not necessarily lead to lower motion estimates and therefore less irradiated tissue. While the motion estimates were different for each fraction, these differences cancel each other out, leading to a non-significantly different value as compared to those motion estimates based on a planning MRI. A daily motion estimate did lead to better inclusion times. For example for Min95, for the same average value of 19–20 mm, the median time that the respiratory signal is within the motion estimate went from 90 to 94%. For this discussion it is very important that one uses an ITV concept to deal with this motion (including the entire motion in the PTV), or adding it to a probabilistic margin recipe (for example the Van Herk recipe [36]). In the latter case uncertainties based only on a planning image are typically viewed as systematic whereas those based on daily imaging are typically viewed as random. Systematic uncertainties are multiplied by a factor 2.5 whereas for random uncertainties this factor is 0.7.

With the daily MRI strategy the respiratory signal of the simulated fractions was outside the estimated motion 6% of the time for Min95, near the 5% that was in the design of the outlier rejection strategy. This suggested that the intrafraction variation was due to outliers and could therefore be mitigated effectively by this outlier rejection strategy. The NoOR has a median of 0% for the same situation. However, to get this 6% improvement, the motion estimate for NoOR was ~ 1 cm larger, corresponding to approximately 30% of the total motion. This could lead to the irradiation of more healthy tissue. Here the trade-off between higher and lower motion estimates (and the underlying choice of applying outlier rejection) could clearly be seen.

**Fig. 4:**
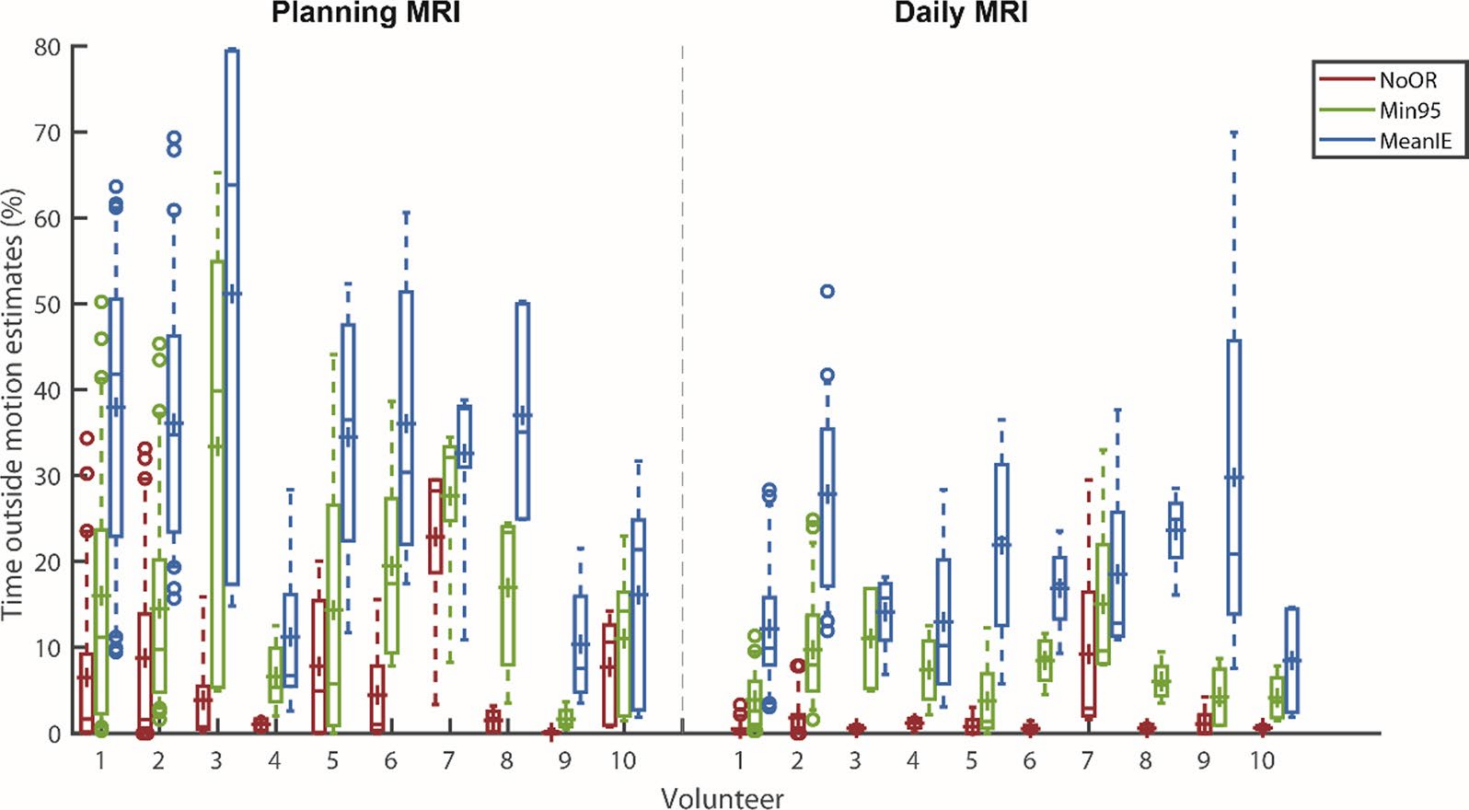
Time outside the motion estimates per volunteer. Relative time outside the motion estimates for NoOR, Min95 and MeanIE for the individual volunteers for the planning MRI (left) and the daily MRI (right) strategies. The percentages were determined per simulated treatment fraction and combined in the boxplots. Boxes: median value (line), mean (cross) and lower and higher quartiles; whiskers: lowest and highest data point in the 9–91% interval, the outliers are indicated by circles. For planning MRI: N = 97 included scans for analysis and N = 10 for constructing motion estimates; for daily MRI strategy N = 71 included scans for analysis and N = 36 for constructing motion estimates

**Fig. 5:**
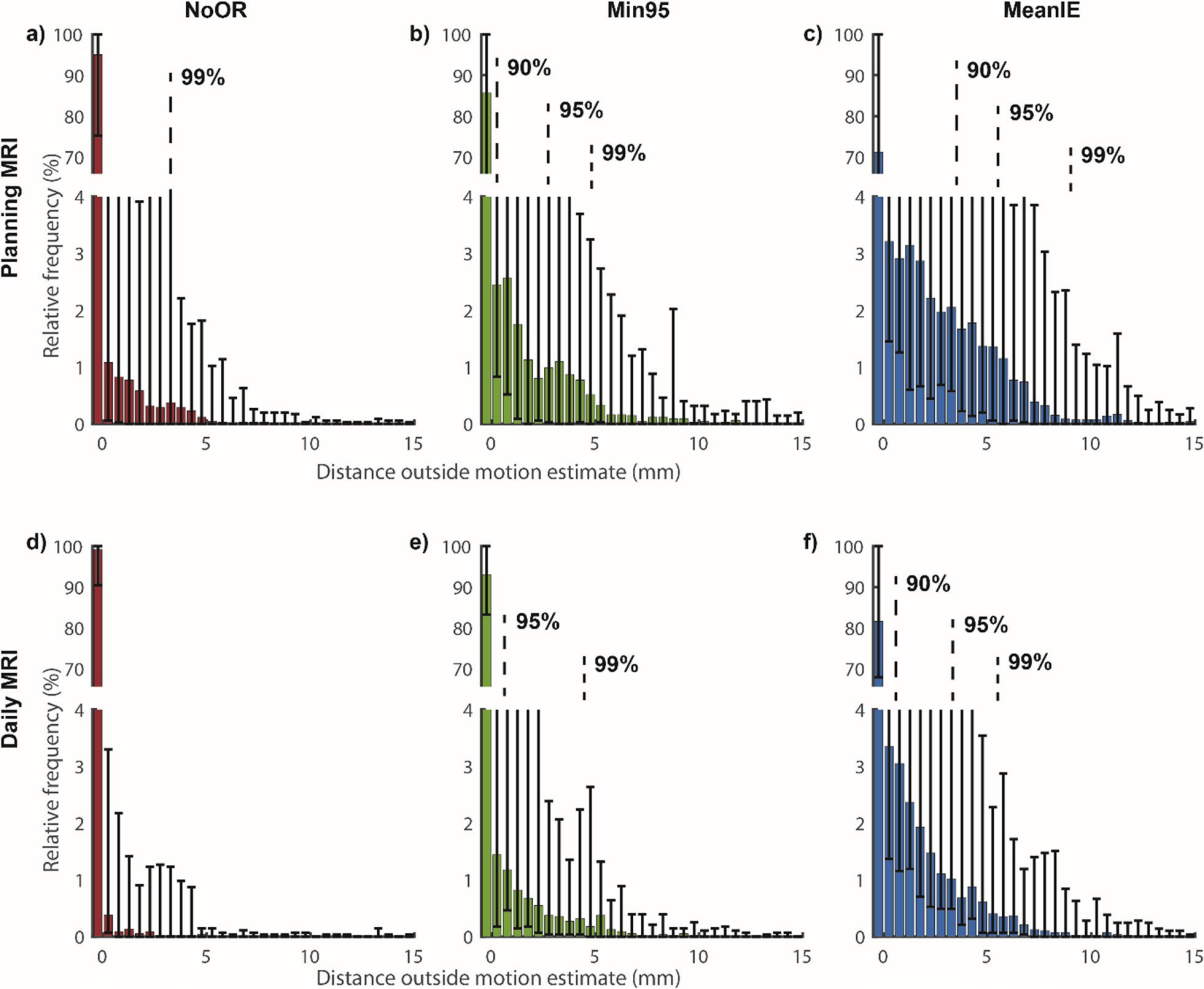
Distances outside motion estimates. Histograms of the relative frequencies the observed motion in the simulated treatment fractions were at a certain distance outside the motion estimates, for planning MRI and daily MRI for NoOR, Min95 and MeanIE. The bars show the median of all volunteers (N = 10) for that distance and the error bars the range. Dotted lines indicate the distances within which 90%, 95% and 99% of the data is included. For planning MRI: N = 97 included scans for analysis and N = 10 for constructing motion estimates; for daily MRI strategy N = 71 included scans for analysis and N = 36 for constructing motion estimates

The time outside the motion estimate (as can be seen in Figs. 3 and 4) could, under simplified assumptions of a uniform dose to a target close to the navigator, be viewed as a surrogate of expected underdosage. If one takes a detailed look at Fig. 5, one can also correlate the time outside the motion estimate to the distance. For example, for the MeanIE strategy using the planning MRI for the motion estimate the signal was outside the estimate for 32% of the time, up to distances of 8 mm (to come to 99% of the data inclusion). Under the simplified assumptions (and not taking any other actions to prevent this) this would mean that the target is receiving less dose than was intended for a third of the treatment fraction. Looking at 5a and 5e one can see that there the effect of the underdosage is comparable (99% of the data being within 3 mm for planning MRI and NoOR which is the most comparable to current clinical practice and within 4 mm for a daily MRI with the Min95 strategy), while motion estimates were significantly smaller for the Daily MRI Min95 strategy. To determine the exact effect of an underestimation of the respiratory signal, a planning study would be necessary. Other research came to a similar conclusion as our work and concluded that inter-fraction variation of breathing makes the use of a single 4D image acquisition not representative for the motion in the abdominal region occurring during the full treatment duration [17, 32, 33]. In the work of Ge et al. [32] and Lens et al. [33], the analysis focused on the mean peak to peak amplitude during a fraction, (a similar analysis of our data can be found in Additional file 1: Fig. A6). However, looking at the time within a fraction as we did for the inclusion time and the distance histograms (Figs. 4 and 5) can give more detailed insights than analyzing single values for entire fractions.

A limitation of our study was that the number of volunteers and sessions was limited. Additionally our subjects were volunteers instead of patients with cancer in the abdominal region. However, patients do not have significantly more regular respiratory motion than healthy volunteers [16] and therefore our conclusions can be expected to also be valid for patients receiving treatment.

Irregular breathing potentially causes sub-optimal radiation treatment (e.g., underdosage of the target volumes, unnecessary irradiation of healthy tissues). 4D strategies for treatment optimization (with or without outlier rejection; with one planning or also a daily image acquisition) may not be the full answer to this problem as suggested by our results. Other strategies should be further investigated. Examples include intrafraction tracking and gating (to deal with the motion) [17, 28, 37], regularization of the breathing motion by conscious mechanical ventilation (to remove the irregularities) [38, 39], and (prolonged) breath-holding (to minimize interfraction and intrafraction organ motion) [39, 40].

## Conclusion

A daily 4DMRI acquisition led to a significantly better estimation of the respiratory motion during the treatment fraction as compared to a single planning 4DMRI acquisition, both with and without outlier rejection strategies. Combining the Min95 technique with a daily pre-beam MRI for the motion estimate led to a small decrease in time inclusion (6%) but could lead to a large decrease in motion that had to be included in the target volume (~ 30%). Outlier rejection did not help in improving the motion estimate of the respiratory motion in case of a single 4D planning image acquisition and often led to an underestimation of the motion. In a radiotherapy setting one has to be aware of this effect also when outlier rejection is used to increase the quality of images as there are possible consequences of such an underestimated motion, for example an underdosage of the target.

## Supplementary Information

**Additional file 1.** This file contains a movie of a 4DMRI, a figure to illustrate the overestimation/underestimation analysis, the values for NoOR, Min95 and MeanIE for the individual patients and scans, the maximum values of the motion for individual patients and scans and an example of one of the volunteers. Finally, an additional analysis is presented on the differences between the motion estimates for planning and daily MRI.

## Data Availability

The datasets generated and/or analyzed during the current study are not publicly available since the participants did not consent in sharing the data with third parties.

## Abbreviations

1D/2D/3D: One-, two-, three-dimensional
4DCT: Four-dimensional computed tomography
4DMRI: Four-dimensional magnetic resonance imaging
CT: Computed tomography
ITV: Internal target volume
MeanIE: Excluding the datapoints of the respiratory signal outside the mean end-inhalation and end-exhalation positions
Min95: Excluding 5% of the respiratory signal whilst minimizing the range
MRgRT: Magnetic resonance guided radiotherapy
MRI: Magnetic resonance imaging
MRL: Magnetic resonance linac
NoOR: No outlier rejection
PTV: Planning target volume
SENSE: Sensitivity encoding
