# Supplementary material for: Evaluating differences in respiratory motion estimates during radiotherapy: a single planning 4DMRI versus daily 4DMRI
Source: Radiat Oncol. 2021 Sep 26;16:188. doi: 10.1186/s13014-021-01915-1 (PMC8474826; doi:10.1186/s13014-021-01915-1)
Supplement: Supplementary file 1 — Additional file 1. This file contains a movie of a 4DMRI, a figure to illustrate the overestimation/underestimation analysis, the values for NoOR, Min95 and MeanIE for the individual patients and scans, the maximum values of the motion for individual patients and scans and an example of one of the volunteers. Finally, an additional analysis is presented on the differences between the motion estimates for planning and daily MRI. [file 13014_2021_1915_MOESM1_ESM.pdf]

Supplemental material

Evaluating differences in respiratory motion estimates during radiotherapy: a single planning 4DMRI versus daily 4DMRI

Duncan den Boer, Johannes K. Veldman, Geertjan van Tienhoven, Arjan Bel, Zdenko van Kesteren

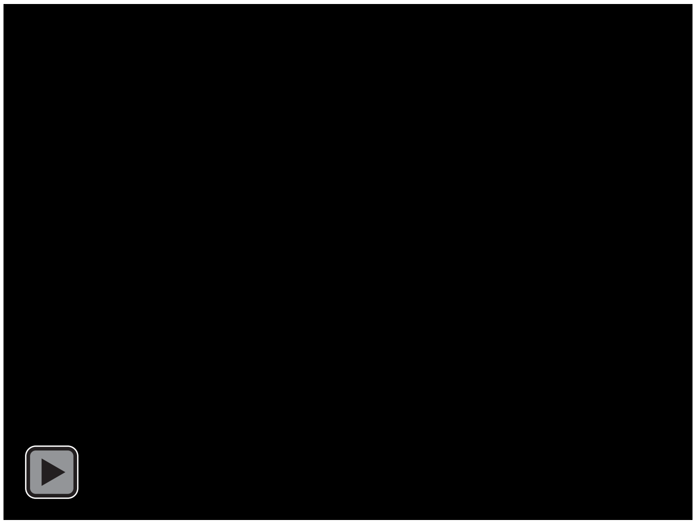

Figure A1. Movie loop of an example 4DMRI.
The ten respiratory states are shown for a single coronal slice, note that this is a representation of the respiratory motion rather than a real breathing cycle, since the images are binned and selected from a 6 minute MR acquisition. If the movie does not play, enable 3D media in Adobe Acrobat Pro: Go to Edit > Preferences > 3D & Multimedia and then select the Enable playing 3D content checkbox.

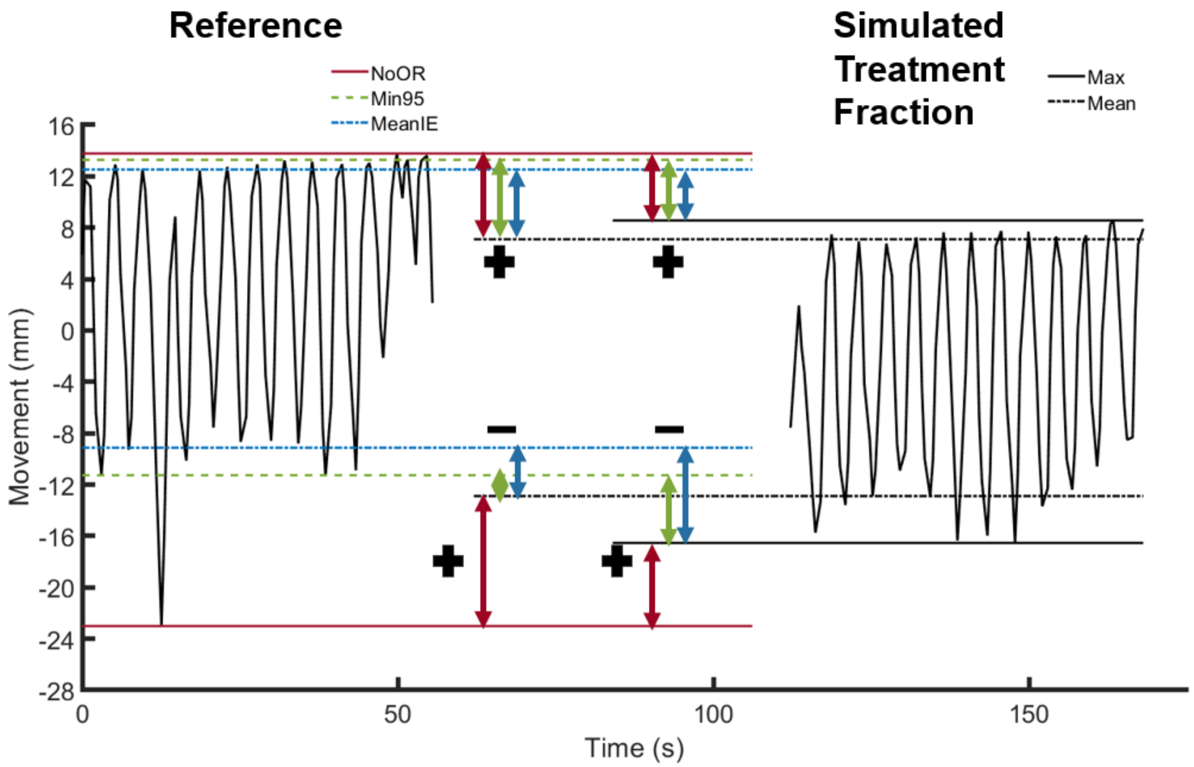

Figure A2. Determining motion estimate overestimation and underestimation.
From the reference scan motion estimates were generated for NoOR, Min95 and MeanIE. The overestimation was defined by this distance to the mean values of the end-inhalation and end-exhalation level (indicated by black dashed line in the figure).
A positive value means that the motion estimate was larger than the observed value during the simulated treatment fraction (indicated by a plus-sign), a negative value means that the motion estimate was smaller (indicated by a minus-sign). Additionally the overestimation was defined with respect to the maximum observed motion during a simulated treatment fraction (indicated by black solid line in the figure). These values were determined both for the end-inhale and end-exhale positions. From these analyses in Fig. A6 were constructed.

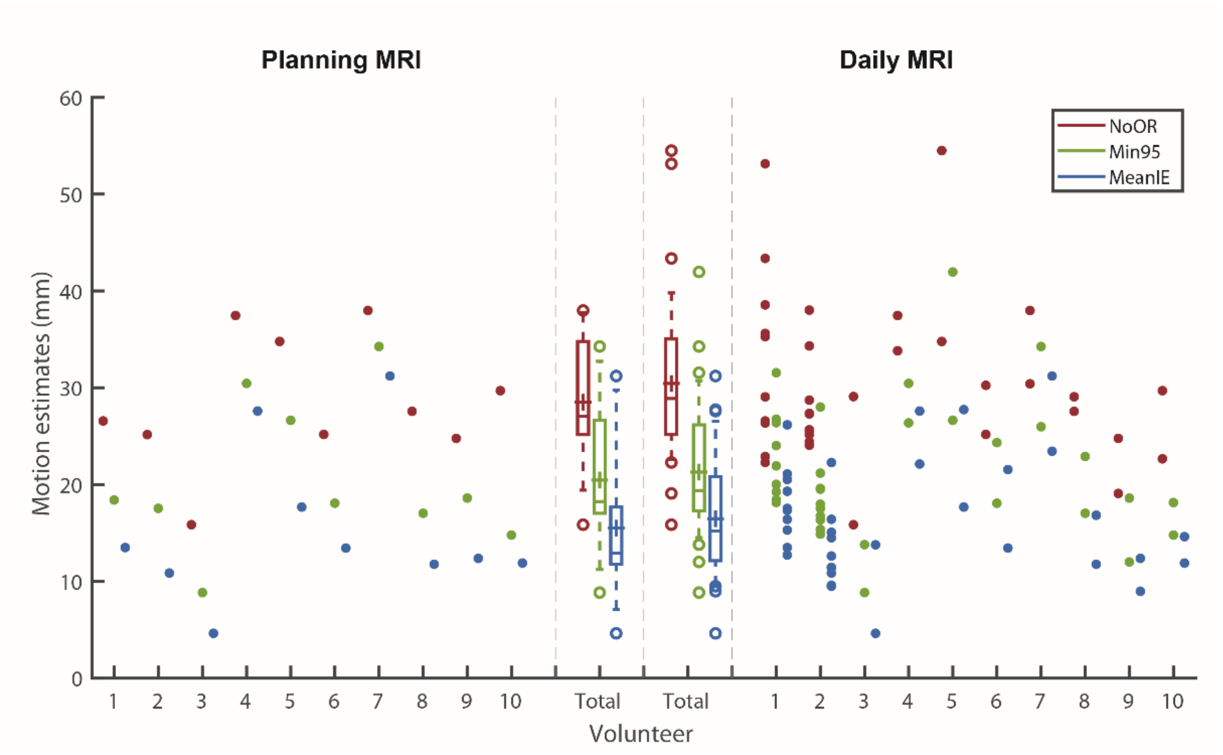

Figure A3. Motion estimates per volunteer.
Left planning MRI, one motion estimate per outlier strategy per volunteer (N=10 per outlier strategy). Right daily MRI; note that there are more motion estimates determined (one per session) per volunteer as can be seen by the number of dots (N = 36 per outlier strategy).

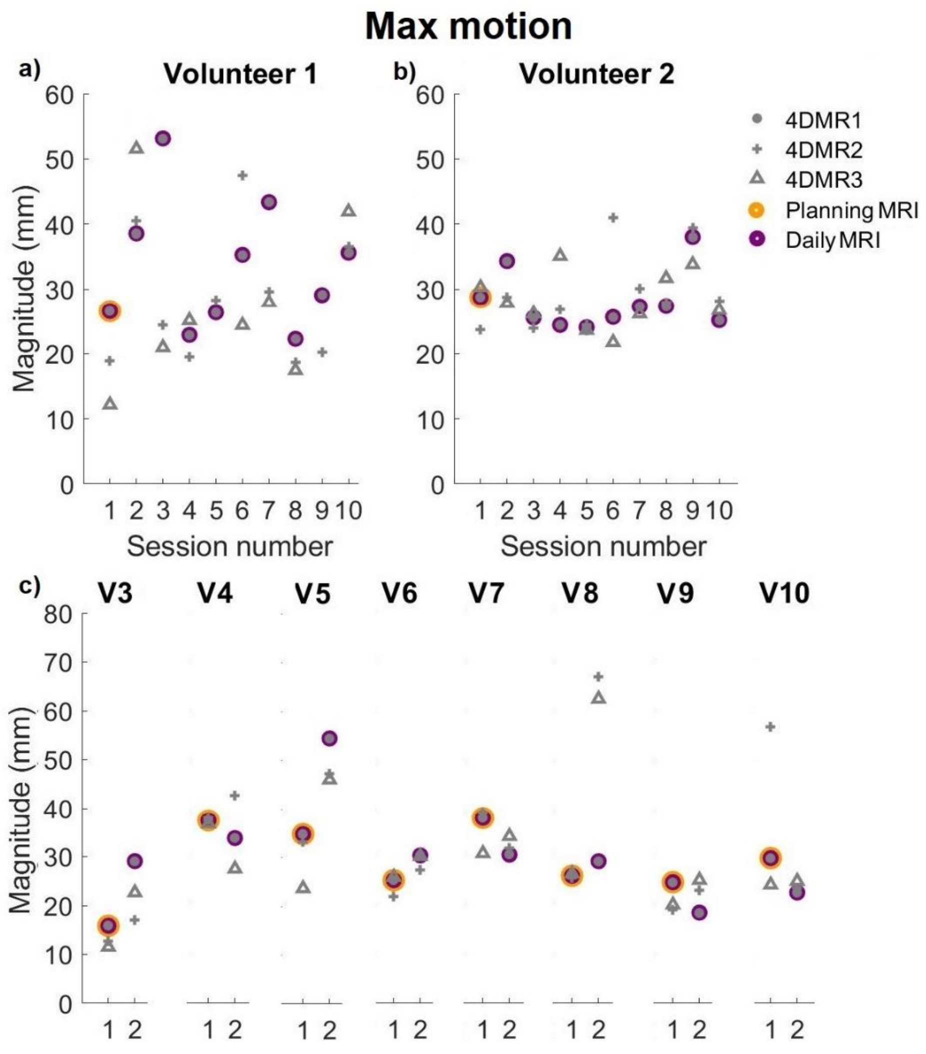

Figure A4. Max motion of all sessions per volunteer.

Maximum motion of each 4DMRI acquisition per session for all volunteers. Volunteer 1 (panel a) and volunteer 2 (panel b) performed 10 sessions with three acquisitions per session. Volunteers 3 through 10 (panel c) each performed 2 sessions with three 4DMRI acquisitions. Daily planning 4DMRIs are denoted using a purple circle around 4DMR1 with 4DMR2 and 4DMR3 as simulated treatment fractions. Single planning 4DMRIs are denoted using an orange circle around 4DMR1 of session 1 with all other acquisitions as simulated treatment fractions.

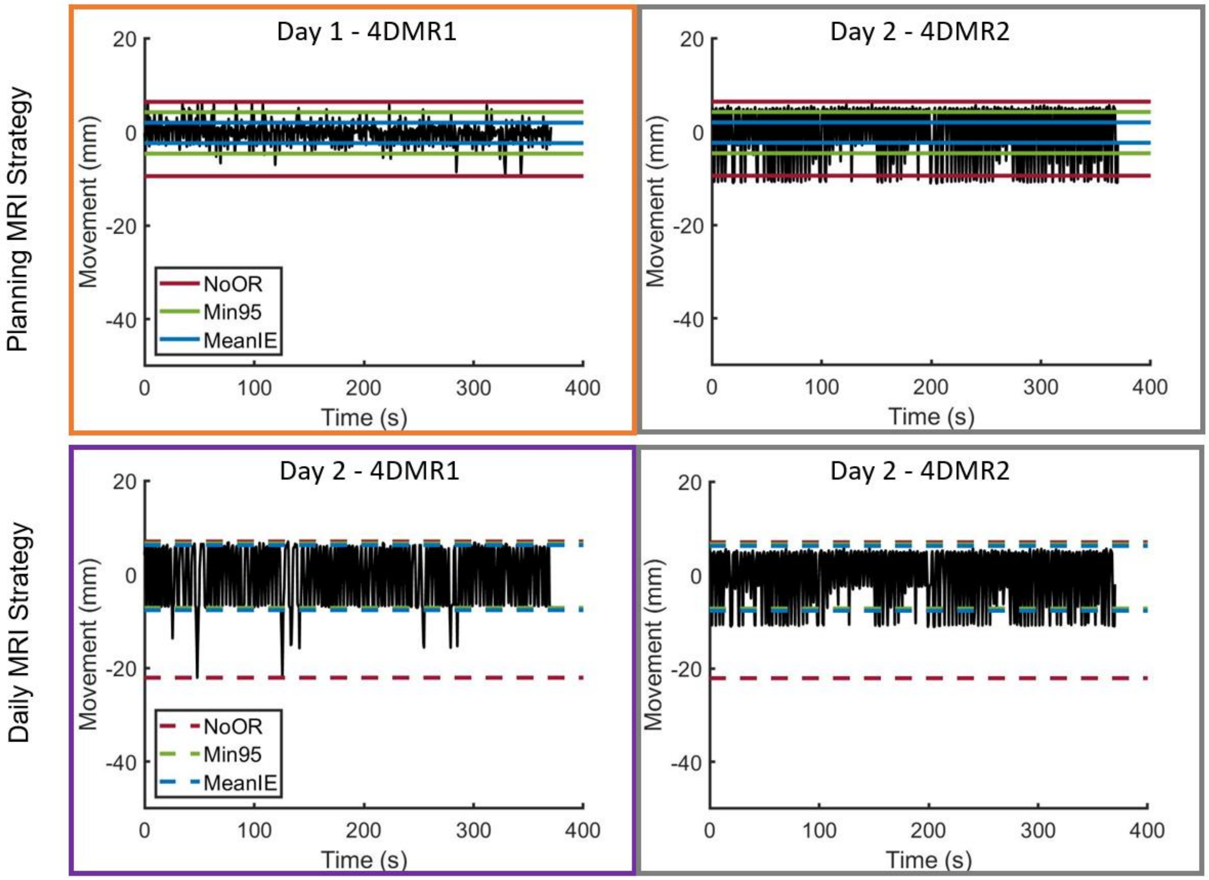

Figure A5. Motion estimates for planning MRI strategy vs. daily MRI strategy for volunteer 3.
Motion estimate using Planning MRI (orange box) causes underestimation in 65% of the time for Min95 and 80% for MeanIE, due to large variation in breathing pattern compared to a subsequent simulated treatment fraction (grey box). Applying the Daily MRI strategy (purple box) decreases this underestimation.

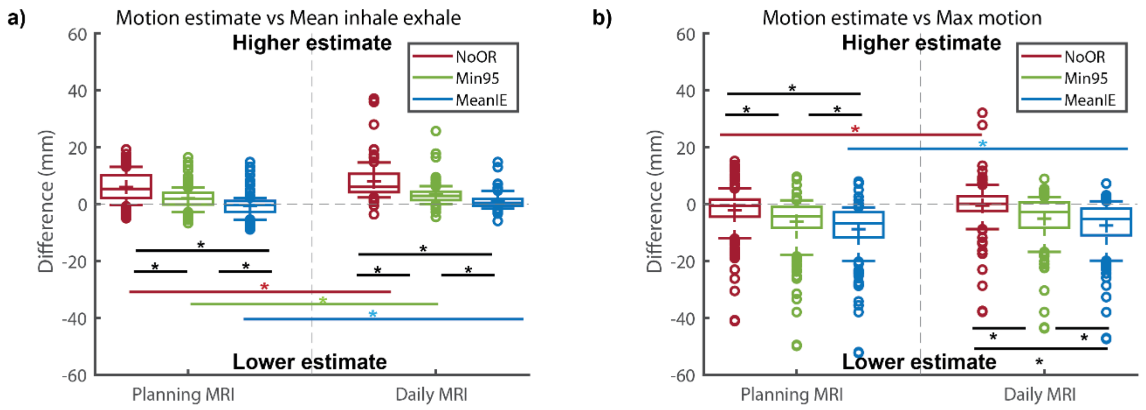

Figure A6. Evaluation of motion estimate magnitude.
*a) Per simulated treatment fraction the distance is determined between the motion estimates for NoOR, Min95 and MeanIE to the mean values of the end-inhalation and end-exhalation level; from these the boxplots were constructed. A positive value means that the motion estimate was larger than the observed value during the simulated treatment fraction, a negative value means that the motion estimate was smaller. For planning MRI strategy N = 97 included scans for analysis and N = 10 for constructing motion estimates; for daily MRI strategy N = 71 included scans for analysis and N = 36 for constructing motion estimates. Boxes: median value (line), mean (cross) and lower and higher quartiles; whiskers: lowest and highest data point in the 9–91% interval, the outliers are indicated by circles. * indicates statistically difference with α = 0.05. b) Similar analysis to (a), but then for the distance between the motion estimate and the maximum values of the end-inhalation and end-exhalation levels during the simulated treatment fraction.*
